# Long-term follow-up of patients with elevated IGF-1 and nadir GH > 0.4 µg/L but < 1 µg/L

**DOI:** 10.1590/2359-3997000000295

**Published:** 2017-09-04

**Authors:** Pedro Weslley Rosario, Maria Regina Calsolari

**Affiliations:** 1 Santa Casa de Belo Horizonte MG Brasil Serviço de Endocrinologia, Santa Casa de Belo Horizonte, MG, Brasil

**Keywords:** Acromegaly, diagnosis, elevated IGF-1, GH suppression

## Abstract

**Objective:**

To report the results of initial investigation and after 5 years of patients with a suspicious clinical scenario for acromegaly, elevated IGF-1, and nadir GH during an oral glucose tolerance test (OGTT) > 0.4 µg/L but < 1 µg/L.

**Subjects and methods:**

Seventeen patients who had elevated IGF-1 (outside puberty and pregnancy) in two measurements and GH between 0.4 and 1 µg/L during OGTT were selected.

**Results:**

During initial assessment, only one patient had microadenoma on magnetic resonance imaging (MRI) of the pituitary. In this patient, IGF-1 returned to normal spontaneously after 5 years. In the remaining 16 patients, spontaneous normalization of IGF-1 was observed in four and IGF-1 continued to be elevated in 12 after 5 years. None of the latter patients developed a phenotype of acromegaly, changes in physiognomy or increase in IGF-1 and no tumor was detected by imaging methods. Two patients had nadir GH < 0.4 µg/L, while the nadir GH remained between 0.4 and 1 µg/L in 10 patients.

**Conclusion:**

In patients (notably young adult or adult women) without a typical phenotype in whom IGF-1 is measured due to a suspicious clinical scenario and is found to be slightly elevated, even if confirmed and in the absence of other causes, a nadir GH cut-off value of 0.4 µg/L instead of 1 µg/L in the OGTT might be inadequate for the diagnosis.

## INTRODUCTION

Untreated acromegaly is associated with higher morbidity and mortality (1-3). The chance of treatment success, which would result in the improvement or reversal of complications, increases if the disease is diagnosed early (1-3). An early diagnosis of acromegaly is therefore desirable and has been encouraged (1-5).

Regarding diagnostic investigation, important points need to be addressed. First, acromegaly is not always accompanied by a typical phenotype as highlighted by some authors: “acromegaly is a clinical syndrome that may not manifest with clear diagnostic features” (1); “some patients with acromegaly have mild or absent clinical features” (2); “we suggest the measurement of IGF-1 in patients without the typical manifestations of acromegaly, but who have several associated conditions” (3), and “the diagnosis does not require the presence of typical phenotypic features” (4). Thus, patients with a suspicious clinical scenario should be investigated even in the absence of a typical phenotype (1-5). Second, while normal IGF-1 virtually excludes the diagnosis of acromegaly (6), elevated concentrations of IGF-1 outside puberty and pregnancy strongly support the hypothesis. Third, the diagnosis of acromegaly is confirmed when elevated IGF-1 is associated with lack of GH suppression during an oral glucose tolerance test (OGTT). In fact, other conditions associated with the lack of GH suppression do not increase IGF-1 but rather reduce it (7). Fourth, magnetic resonance imaging (MRI) of the pituitary should be obtained, but the absence of adenoma on MRI does not rule out the diagnosis of acromegaly as stated by some authors: “occasionally patients will not have imaging evidence of a pituitary adenoma” (8); “some patients with acromegaly have small or undetectable tumour” (2), and “the diagnosis is a biochemical one and does not require the presence of a pituitary tumor on MRI” (4).

A GH cut-off value of 1 µg/L in the suppression test has traditionally been used for the diagnosis of acromegaly (9). However, patients with acromegaly and GH (basal or nadir) concentrations < 1 µg/L are not uncommon (2,10,11). At present, most authors consider nadir GH levels > 0.3 µg/L or 0.4 µg/L during OGTT sufficient for the diagnosis of acromegaly in patients with elevated IGF-1 (1,4,12-18). Although increasing sensitivity, it is important to evaluate whether this cut off does not lead to unnecessary investigations, equivocal diagnoses and, consequently, treatments that are not indicated. It should be remembered that, in opposition to reducing the cut off, the Endocrine Society still considers GH < 1 µg/L in the OGTT sufficient for exclusion of acromegaly (3).

The objective of this study was to report the results of initial investigation and after 5 years of patients with a suspicious clinical scenario (1-5) and elevated IGF-1, who would have a diagnosis of acromegaly based on the nadir GH cut-off value of 0.4 µg/L (1,4,12-18) but not 1 µg/L (3,9).

## SUBJECTS AND METHODS

### Patients

First, 4,350 adults (age between 18 and 70 years, excluding pregnant women and patients with known pituitary disease) underwent acromegaly screening: 2,270 patients with type 2 diabetes mellitus or glucose intolerance (19), 178 patients who reported “enlargement of their extremities” (20), and 1,902 patients with two or more comorbidities related to acromegaly [including arterial hypertension in 1,806 patients (21)]. In patients with elevated IGF-1, a new measurement was obtained and was combined with the measurement of GH during an OGTT. For this study, patients with a suspicious clinical scenario (1-5) according to the definition below (1,3,5), who had a diagnosis of acromegaly (i.e., elevated IGF-1 in two measurements outside puberty and pregnancy associated with lack of GH suppression during OGTT) based on the cut-off value of 0.4 µg/L (1,4,12-18) but not 1 µg/L (3,9), were selected. The study and its respective protocol were approved by the Ethics Committee of our institution.

### Definitions

A typical acromegalic phenotype was defined i) by an endocrinologist with experience in the disease (P.W.R.), ii) based on ectoscopy, and iii) considering acral enlargement and maxillofacial changes (3).

A suspicious clinical scenario was defined in the presence of two or more comorbidities related to acromegaly according to the Canadian Consensus (5), American Association of Clinical Endocrinologists (1), and Endocrine Society (3). The comorbidities considered were (1,3,5): i) nonspecific chronic headache (for example, migraine and hypertensive headache were not considered); ii) generalized and persistent excessive sweating; iii) diffuse arthralgias associated with some radiologic alteration (22) in the absence of known rheumatological disease (reported by the patient, suspected, or confirmed in the medical record); iv) chronic fatigue not explained by any other underlying disease (among the diagnoses reported by the patient or present in the medical record); v) bilateral paresthesias (Carpal tunnel syndrome); vi) recently diagnosed diabetes mellitus; vii) recently diagnosed arterial hypertension requiring antihypertensive medication.

### Follow-up

During initial assessment, the patients were submitted to MRI of the pituitary using gadolinium as contrast agent. Patients without adenoma on MRI underwent chest and abdominal contrast-enhanced computed tomography (CT). These patients were not treated for acromegaly. The patients were reevaluated clinically and by laboratory testing (serum IGF-1) after 5 years. The presence of a typical phenotype (see above) and changes in physiognomy were evaluated by comparing current photographs and those obtained at the time of initial assessment. Patients with persistently elevated IGF-1 were submitted to a new GH suppression test and 3-tesla MRI of the pituitary (23) using gadolinium as contrast agent. Patients with elevated IGF-1, in the absence of GH suppression and adenoma on MRI, were again submitted to chest and abdominal CT.

The samples were collected in the morning after an approximately 10-h fast, with the subject resting for 20 min before and during the OGTT. For the OGTT, GH was measured before and 30, 60, 90 and 120 min after the oral administration of 75 g anhydrous glucose.

GH was measured with a chemiluminescence assay (Immulite, Diagnostic Products Corporation, Los Angeles, CA) with an analytical sensitivity ≤ 0.05 µg/L. The standard provided by the kit was calibrated against the World Health Organization (WHO) 2^nd^ International Standard (IS) 98/574. The results are expressed as µg/L. IGF-1 was also measured with a chemiluminescent assay (Immulite 2000, Diagnostic Products Corporation, Los Angeles, CA) (analytical sensitivity of 25 μg/L) using antibodies highly specific for IGF-1 and previously established reference values stratified by age based on a sample of 1,000 subjects rigorously selected in the same town where the study was conducted (24). “Functional separation” (acidification followed by saturation with IGF-2) was the technique used to exclude interference from IGF-binding proteins (IGFBPs).

We highlight that on initial assessment the measurements were made before the period in which overestimated IGF-1 values began to be observed (25-27). On last assessment, the measurements were made in 2015 using lots that, according to the manufacturer (25) and in our laboratory (28), are in alignment with the medians of the reference range data published in the Instructions For Use.

## RESULTS

The study included 16 women and one men aged 30 to 55 years (median 41 years). Initial IGF-1 ranged from 1.08 to 1.53 times the upper limit of the normal range (ULN) for age (24). The frequency of comorbidities is showed in the Table 1. On initial assessment and 5 years later, none of the patients had kidney or liver failure or malnutrition, or was using oral estrogen. Seven patients had diabetes mellitus, but were compensated (29) at the time of IGF-1 measurement and OGTT. Thyroid dysfunction and pregnancy (in premenopausal women) were excluded in all patients.

**Table 1:** Frequency of comorbidities

Comorbidity	Number of patients (%)
Recently diagnosed arterial hypertension	15 (88.2%)
Recently diagnosed diabetes mellitus	12 (70.6%)
Nonspecific chronic headache	10 (58.8%)
Bilateral paresthesias (carpal tunnel syndrome)	9 (53%)
Generalized and persistent excessive sweating	6 (35.3%)
Chronic fatigue not explained by any other underlying disease	6 (35.3%)
Diffuse arthralgias with some radiologic alteration in the absence of known rheumatological disease	5 (29.4%)

During initial assessment, a lesion suggestive of microadenoma was detected in one patient by MRI of the pituitary (hypointense nodule measuring 4 mm in diameter and showing no contrast enhancement after the administration of gadolinium) (Table 2). Other hormone hypersecretions were excluded in this patient. The woman was not submitted to surgical or medicamentous treatment for acromegaly based on the absence of a typical phenotype, low GH concentrations (nadir < 1 µg/L during OGTT), and good control of comorbidities with conventional treatments. After 5 years, the patient exhibited no changes in physiognomy, remained without a phenotype, and had normal IGF-1 (confirmed in two measurements). MRI was repeated in this case to exclude tumor apoplexy and the lesion was found to be unchanged.

**Table 2:** Results of the patients with microadenoma on MRI

	Initial assessment	Last assessment
Sex	Female	
Age	50 years	56 years
Clinical scenario	Carpal tunnel syndrome, hypertension, headache, dyslipidemia, glucose intolerance	Hypertension, dyslipidemia, glucose intolerance
Serum IGF-1	1.28 x ULN	0.9 x ULN
Nadir GH	0.65 µg/L	Not performed
MRI	Microadenoma (4 mm)	Microadenoma (4 mm)

ULN: upper limit of normal range; MRI: magnetic resonance imaging; DM: diabetes mellitus; GI: glucose intolerance.

MRI did not detect adenoma or pituitary enlargement, and chest and abdominal CT did not reveal a tumor during initial assessment in 16 patients. After 5 years, these patients did not develop changes in physiognomy and remained without a phenotype. IGF-1 spontaneously returned to normal in 4 patients (confirmed in two measurements) and elevated IGF-1 persisted in 12.

Regarding the 12 patients with persistently elevated IGF-1, the last IGF-1 ranged from 1.1 to 1.61 times the ULN, already considering the current age of the patient. Comparing the final and initial concentrations, none of the patients exhibited a significant increase in IGF-1, i.e., increment > 20% [limit defined based on the variation found in 100 healthy subjects rigorously selected and in stable conditions, who were submitted to IGF-1 measurement at an interval of 3 months using the same assay as employed in this study (24)]. In a new OGTT, GH suppression was achieved in 2 patients and 10 continued with nadir GH between 0.4 and 1 µg/L. MRI of the pituitary (obtained for all patients) and chest and abdominal CT (obtained for the 10 patients without GH suppression) again revealed no tumor.

Thus, 7 patients no longer had a diagnosis of acromegaly (based on spontaneous normalization of IGF-1 in 5 and on GH suppression in 2). None of the 10 patients with persistently elevated IGF-1 and nadir GH > 0.4 µg/L after 5 years developed a phenotype of acromegaly, changes in physiognomy or increase in IGF-1 and no tumor was detected by the imaging methods.

## DISCUSSION

There is consensus that not only patients with typical phenotypic features should be investigated for acromegaly (1-5). Although not presenting the typical acromegalic phenotype, the patients included in this study had two or more comorbidities commonly found in “active” acromegaly (1,3,5), and additional criteria were required to consider them compatible with this condition (see Subjects and Methods). Moreover, the age range of our patients (30-60 years) coincides with that of a higher incidence of the disease. Consequently, there was a suspicious clinical scenario justifying investigation for acromegaly (1-5).

Elevated IGF-1 does not always indicate acromegaly, but its specificity increases when measured outside puberty and pregnancy (situations characterized by physiological elevation of this hormone). Furthermore, the results should be confirmed in a subsequent measurement. One cause of falsely elevated IGF-1 are inadequate limits of normality. When defined using an inadequately selected sample or an insufficient number of subjects, the upper limit may be underestimated and, consequently, an individual with normal IGF-1 would be erroneously classified as having elevated IGF-1. In the present study, the definition of elevated IGF-1 was based on the limits established for a sample of 1,000 subjects from the same town as the patients included in this study. This sample was selected rigorously (exclusion of interfering conditions and medications and extremes of body mass index) and stratified by decade of life (24) according to current recommendations. Hence, in the present study “elevated IGF-1” refers to the measurement obtained outside puberty and pregnancy, confirmed in two measurements, and based on adequate normative information. Although theoretically possible, heterophile antibodies are not cited as possible agents that interfere with serum IGF-1. Moreover, the only case report in the literature mentioning interference of these antibodies with the Immulite assay inexplicably found a reduction in IGF-1 (30). The assay used does not show cross-reactivity to insulin or IGF-II and eventual interference from IGFBPs would cause a reduction in IGF-1 (24). Overweight/obese subjects have higher hepatic sensitivity to GH. However, there is no elevation of serum IGF-1 (31). It has also been suggested that genotype d3 of the GH receptor (d3-GHR) increases sensitivity to this hormone (32). However, to our knowledge, there is no study reporting an association between the presence of d3-GHR and elevated IGF-1 in individuals without acromegaly and not treated with GH.

The diagnosis of acromegaly is made when elevated IGF-1 is associated with the “absence of GH suppression” during an OGTT. In fact, other conditions that can cause a lack of GH suppression do not result in IGF-1 elevation, but rather reduce it (7). Nevertheless, these conditions were excluded in our patients. Most authors define a nadir > 0.4 µg/L as “lack of GH suppression” (1,4,12-18) and many others recommend even lower cut offs, 0.3 µg/L (4,12,14,15,17), 0.25 µg/L (13), and 0.2 µg/L (16).

Since the patients of the present study had i) “a suspicious clinical scenario” (1-5); ii) “elevated IGF-1” in two measurements and excluding other causes; iii) “lack of GH suppression” during OGTT using a cut off accepted by most authors (1,4,12-18), and iv) considering that “the diagnosis is a biochemical one and does not require the presence of a pituitary tumor on MRI” (4) since “some patients with acromegaly have small or undetectable tumour” (2) and “occasionally patients will not have imaging evidence of a pituitary adenoma” (8), they could have been diagnosed with acromegaly during initial assessment and submitted to medicamentous treatment or exploratory transsphenoidal surgery (8). However, follow-up suggests that these patients probably did not have acromegaly, First, considering the interval between the onset of manifestations and diagnosis, with the typical phenotype already present (33,34), the absence of this phenotype and of changes in physiognomy after 5 years makes the disease unlikely. Second, spontaneous normalization or absence of an increase in IGF-1 after this period also weakens the diagnosis. Even in patients with persistently elevated IGF-1, GH concentrations in the OGTT continued to be < 1 µg/L. Third, 3-tesla MRI of the pituitary (23) and chest and abdominal CT were negative in the initial and last assessment (in patients in whom biochemical alterations persisted).

There is no question that the recommendation of investigating acromegaly in patients with a suspicious clinical scenario, even in the absence of a typical phenotype (1-5), is interesting for early diagnosis. However, since comorbidities associated with acromegaly are also common in the adult population (*e.g*., diabetes mellitus, arterial hypertension, carpal tunnel syndrome, nonspecific headache), it is expected that this investigation does not confirm the disease in most patients (low pre-test probability). By definition, approximately 2% of the normal population has “elevated IGF-1”. Obviously, the concentrations in these individuals do not deviate much from the ULN, but IGF-1 is also not very elevated in patients with acromegaly and nadir GH between 0.4 and 1 µg/L [up to 2/3 have IGF-1 ≤ 2 x ULN (2,10,11)], and the intensity of IGF-1 elevation is therefore little useful for this distinction. Although increasing sensitivity, the reduction in the nadir GH cut off from 1 µg/L to 0.4 µg/L may decrease the specificity of the diagnostic criterion. Indeed, even after the exclusion of interfering conditions and using sensitive assays calibrated against the second IS 98/574 for hGH, normal individuals, notably young adult and adult women, may have nadir GH > 0.4 µg/L in the OGTT (6,35-38). In the present series, all but one patient were women ≤ 55 years. Thus, the high prevalence of comorbidities associated with acromegaly in the general adult population, the fact that even individuals without disease can have slightly elevated IGF-1 and nadir GH > 0.4 µg/L in the OGTT and the rarity of acromegaly may explain why even the combination of findings (suspicious clinical scenario (1-5), elevated IGF-1, nadir GH > 0.4 µg/L) can have a low positive predictive value.

In patients without a typical phenotype, notably young adult or adult women, in whom IGF-1 is measured due to a suspicious clinical scenario (1-5) and is found to be slightly elevated, even if confirmed and in the absence of other causes, a GH cut-off value of 0.4 µg/L (1,4,12-18) instead of 1 µg/L (3) in the OGTT might be inadequate for the diagnosis of acromegaly. We do not know whether these individuals correspond to the portion of the “normal” population that exhibits concentrations outside the reference range or have GH hypersecretion, although not tumoral. Additionally, we do not know whether these persistently elevated concentrations of IGF-1 increase the risk of comorbidities despite the absence of acromegaly, remembering that all of these patients had comorbidities. Since GH hypersecretion should be investigated in all patients with pituitary incidentaloma, even in the absence of a phenotype (3,4,39), the conclusion of the present study may also have implications for the diagnosis of clinically silent somatotropinoma, remembering that this diagnosis could change expectant management (in the case of non-functional incidentaloma) to surgery or medicamentous treatment (in the case of somatotropinoma) (39).
